# 

**Published:** 1892-02

**Authors:** 


### CONTENTS:

Article I.—Minutes of the Twenty-third Annual Session of the Southern Dental Association. - - - - - 433

" II.—Antiseptics and Disinfectants used in Dental Surgery. By Savill H. Hayward, - - - - - - - 455

" III.—Peroxide of Hydrogen as a Therapeutical and Bleaching Agent. By Dr. P. P. Starke, - - - - 459

" IV.—Alveolotomy. By G. S. Junkerman, M. D., D. D. S. - 469

### BIBLIOGRAPHICAL.

Dental Medicine, - - - - - - - - 472

### MONTHLY SUMMARY.

Tumenol, - - - - - - - - - 472

Chromic Acid in Syphilitic Affections of the Mouth, - - - 474

A New Styptic, - - - - - - - - 476

Phenocoll Hydrochlorate, - - - - - - - 477

Enlargement of the Cervical Glands, Due to the Impaction of Lower Left Wisdom Tooth. - - - - - - 478

A Case in Practice, - - - - - - - - 479

Ulcers and Gangrenous Wounds. - - - - - - 480

A Convenient Method of Adding New Teeth to Old Plates. - - 480

